# Comparative Adverse Event Reporting Patterns of Amoxicillin and Amoxicillin/Clavulanic Acid in FAERS: A 2022–2025 Disproportionality Analysis

**DOI:** 10.1002/hsr2.73328

**Published:** 2026-10-05

**Authors:** Pierangela Irrera, Ilaria Ammendolia, Gabriella Frattima, Fabiola De Luca, Michela Campolo, Emanuela Esposito, Alessio Ardizzone, Fabrizio Calapai

**Affiliations:** ^1^ Department of Chemical Biological, Pharmaceutical and Environmental Sciences, University of Messina Messina Italy; ^2^ UniCamillus‐Saint Camillus International University of Health Sciences Rome Italy; ^3^ Department of Biomedical Sciences Dental Sciences and Morpho‐Functional Imaging, University of Messina Messina Italy

**Keywords:** amoxicillin, amoxicillin/clavulanic acid, antimicrobial effectiveness, FAERS, pharmacovigilance

## Abstract

**Background and Aims:**

Amoxicillin, alone or combined with clavulanic acid, is widely used for the treatment of bacterial infections. While clavulanic acid expands amoxicillin's antibacterial spectrum by inhibiting β‐lactamases, differences in their real‐world safety profiles and reporting patterns remain relevant for pharmacovigilance and antimicrobial surveillance. This study aimed to characterize and compare adverse event reporting associated with amoxicillin and amoxicillin/clavulanic acid and to explore reporting signals involving Preferred Terms potentially associated with reduced treatment effectiveness.

**Methods:**

Adverse drug reaction reports recorded in the FDA Adverse Event Reporting System (FAERS) Public Dashboard between 2022 and 2025 were analyzed. Disproportionality analyses were performed at the MedDRA Preferred Term level using Reporting Odds Ratios, Proportional Reporting Ratios, and Information Component‐based methods. Selected Preferred Terms were further evaluated through direct comparison between the two therapies.

**Results:**

Amoxicillin was predominantly associated with hypersensitivity and cutaneous adverse event signals, whereas amoxicillin/clavulanic acid showed a stronger hepatobiliary reporting profile, including signals for drug‐induced liver injury, cholestasis, and hepatic cytolysis. Direct comparison confirmed distinct reporting patterns, with relatively stronger liver‐related signals for the combination therapy and stronger hypersensitivity‐related signals for amoxicillin alone. Preferred Terms potentially associated with reduced treatment effectiveness or antimicrobial resistance were identified in both groups; however, these reporting signals may reflect heterogeneous clinical scenarios and should not be interpreted as evidence of confirmed therapeutic failure or microbiological resistance.

**Conclusion:**

These findings support the use of pharmacovigilance databases as complementary tools for antibiotic safety monitoring and for identifying reporting patterns that may warrant further investigation, including reports suggestive of reduced treatment effectiveness. However, such signals should be interpreted cautiously, as they do not establish reduced antimicrobial effectiveness or antimicrobial resistance and may reflect a variety of underlying clinical and therapeutic factors.

## 1 Introduction

Amoxicillin is a semisynthetic aminopenicillin obtained through the introduction of an additional amino group into the penicillin core structure, a modification originally designed to improve oral absorption and expand antibacterial activity [1]. Structurally, amoxicillin contains a central β‐lactam ring and a p‐hydroxyphenylglycylamino side chain, conferring enhanced pharmacokinetic properties and a broader antibacterial spectrum compared with earlier penicillins [2]. As a β‐lactam antibiotic, amoxicillin exerts its bactericidal effect by irreversibly binding to penicillin‐binding proteins, particularly penicillin‐binding protein 1A, which plays a critical role in bacterial cell wall synthesis [3]. Inactivation of this protein ultimately leads to inhibition of peptidoglycan cross‐linking and bacterial cell lysis [3].

Owing to its efficacy, favorable safety profile, and extensive clinical experience, amoxicillin, either administered alone or in combination with clavulanic acid, has become the most frequently prescribed penicillin in Europe and in many other regions worldwide [4]. Clinically, amoxicillin is indicated for the treatment of infections caused by β‐lactamase–negative bacteria, including upper and lower respiratory tract infections, urinary tract infections, skin and soft tissue infections, and as part of eradication regimens for Helicobacter pylori [5]. However, the widespread use of amoxicillin has inevitably contributed to the emergence and dissemination of β‐lactamase‐producing organisms, resulting in reduced susceptibility and therapeutic failure [6].

To address this limitation, clavulanic acid was introduced as a β‐lactamase inhibitor capable of restoring amoxicillin activity against resistant pathogens [7]. Clavulanic acid is a β‐lactam‐oxazolidine compound characterized by an azabicyclo[3.2.0]heptane ring system, which enables irreversible binding to the active site of numerous β‐lactamases, leading to enzyme inactivation [8]. Although clavulanic acid exhibits minimal intrinsic antibacterial activity, its combination with amoxicillin preserves the antibiotic's established effectiveness against β‐lactamase–negative strains while restoring and expanding activity against β‐lactamase‐producing organisms [9]. This combination has demonstrated activity against a range of pathogens, including anaerobes such as Bacteroides fragilis infection, and may be used in selected urinary, respiratory, and soft tissue infections [10, 11, 12]. However, in light of evolving resistance mechanisms, its role in the treatment of infections caused by resistant organisms, including Klebsiella pneumoniae infection, is now limited and it is generally not considered a first‐line option, its use should therefore be guided by microbiological data and current clinical guidelines [10, 11, 12].

Both amoxicillin and amoxicillin/clavulanic acid are generally regarded as well‐tolerated agents with a long‐standing record of clinical safety. Nevertheless, adverse drug reactions (ADRs) are well documented. Hypersensitivity reactions represent a major concern for amoxicillin and preclude its use in patients with confirmed penicillin allergy [13, 14]. In contrast, the combination therapy has been more frequently associated with hepatic adverse events, including cholestatic jaundice and hepatocellular injury, particularly in elderly patients and those with pre‐existing liver disease [15]. For this reason, amoxicillin/clavulanic acid should be used with caution in patients with hepatic impairment and is contraindicated in individuals with a history of cholestatic jaundice or hepatic dysfunction related to previous exposure [16]. Molecular and chemical mechanisms underlying β‐lactam‐associated hepatotoxicity and multidrug resistance have been previously described, highlighting the complex interplay between drug structure, metabolism, and bacterial adaptive responses [17, 18].

In parallel with safety considerations, antimicrobial resistance has emerged as a major global public health challenge and represents an increasingly important contributor to reduced antibiotic effectiveness [19]. Despite its clinical relevance, antimicrobial resistance is often underrecognized in pharmacovigilance frameworks, where traditional ADRs tend to receive greater attention. Treatment failure or drug inefficacy, however, may represent an indirect but meaningful signal of emerging resistance, particularly when observed across large populations and extended periods of time.

Pharmacovigilance databases, which systematically collect spontaneous reports of suspected ADRs from healthcare professionals, patients, and regulatory authorities, offer a unique opportunity to investigate real‐world safety issues and potential resistance‐related outcomes. Nevertheless, the use of such databases to explore antimicrobial resistance remains limited. The existing literature reveals a scarcity of studies leveraging real‐world pharmacovigilance data to evaluate adverse reactions and resistance signals associated with amoxicillin and amoxicillin/clavulanic acid, particularly within large spontaneous reporting systems [20]. It has been proposed that antimicrobial resistance itself may be conceptualized as an overlooked adverse outcome and that pharmacovigilance databases could serve as valuable tools for monitoring antibiotic utilization patterns and resistance trends at a population level [21].

In this context, the present study was undertaken to investigate resistance‐related signaling within spontaneous adverse reaction reports and to evaluate the potential role of pharmacovigilance databases in supporting antimicrobial resistance surveillance. Specifically, unsolicited reports of suspected adverse reactions to amoxicillin and amoxicillin/clavulanic acid recorded in the FDA Adverse Event Reporting System (FAERS) between 2022 and 2025 were analyzed, with particular emphasis on reports of drug inefficacy and their possible implications as indicators of emerging antimicrobial resistance. Indeed, despite FAERS was primarily developed for post‐marketing safety surveillance, its large‐scale real‐world reporting data may also provide hypothesis‐generating signals related to therapeutic performance. Such signals should be interpreted cautiously, as they do not constitute direct evidence of antimicrobial resistance and require independent clinical and microbiological validation.

## 2 Methods

### 2.1 Data Source, Case Selection, and Deduplication

We used the FDA Adverse Event Reporting System (FAERS) quarterly data files for 16 consecutive quarters, from 2022Q1 through 2025Q4. The files were accessed on 20 April 2026. All four quarterly public releases for 2025 were available and included in the analysis.

FAERS is a spontaneous reporting database used for post‐marketing safety surveillance, and publicly available quarterly data files were used for the present analysis (FDA FAERS Public Dashboard FAQ; FDA FAERS Public Dashboard About). Reports involving amoxicillin or the fixed‐dose combination of amoxicillin/clavulanic acid were identified across the quarterly datasets through generic names and commonly reported textual variants. To avoid double counting between exposure groups, exposure assignment was performed hierarchically at the PRIMARYID level. Reports containing evidence of both amoxicillin and clavulanate were classified as amoxicillin/clavulanate and were not simultaneously counted in the amoxicillin‐only cohort [22].

### 2.2 Disproportionality Analysis

Adverse events were analyzed at the MedDRA Preferred Term (PT) level. Disproportionality was assessed using Reporting Odds Ratios (RORs) with 95% confidence intervals, Proportional Reporting Ratios (PRRs), and chi‐square statistics based on standard case–non‐case methodology against the FAERS background database [23]. A signal was considered present when the lower limit of the 95% confidence interval of the ROR exceeded 1. For PRR, a signal was considered present when at least 3 reports were available for the PT, the PRR was at least 2, and the chi‐square value was at least 4, according to commonly used signal detection criteria [24]. To complement the frequentist approach, a Bayesian analysis based on the Information Component (IC) was also performed, and Bayesian support was considered present when the lower credibility bound of the IC exceeded 0 [25]. A direct head‐to‐head comparison between amoxicillin and amoxicillin/clavulanic acid was additionally carried out for selected PTs [26].

### 2.3 Secondary and Sensitivity Analyses and FAERS Detailed Protocol

Several secondary and sensitivity analyses were performed to assess the robustness and clinical consistency of the main findings. First, the disproportionality analysis was repeated after restricting the dataset to reports in which the study drug was classified as primary suspect only. Second, selected clinically relevant PTs were further explored according to sex and age. Age values reported in different units were standardized in years whenever possible and then grouped as < 18 years, 18–64 years, and ≥ 65 years. Third, an exploratory analysis according to concomitant‐drug burden was performed to examine whether the main PT signals remained detectable across reports with different levels of therapeutic complexity. In addition, selected PTs were grouped into clinically interpretable categories, including hypersensitivity‐related, hepatobiliary, renal, therapeutic ineffectiveness, and resistance‐related terms, in order to facilitate narrative interpretation of the signal pattern. All data processing and statistical analyses were performed in RStudio. Since FAERS data are publicly available and anonymized, ethical approval and informed consent were not required.

In detail, drug exposures were identified from the FAERS DRUGNAME and PROD_AI fields. Text was converted to upper case, trimmed, and normalized for internal whitespace. Amoxicillin evidence was identified using the marker AMOXIC, while clavulanate evidence was identified using CLAV, CLAVULAN, AUGMENT, or CO‐AMOX. Classification was then applied at the report (PRIMARYID) level. The amoxicillin/clavulanate group required evidence for both components, either in a fixed‐combination expression or as separate eligible components in the same report. Amoxicillin‐containing multicomponent products without clavulanate were excluded from the strict amoxicillin‐only comparative cohort. The complete dictionary of captured expressions and their classification rules is provided in Supporting Information S1: Table S1.

Moreover, exposure groups were assigned hierarchically at the PRIMARYID level. Evidence for both amoxicillin and clavulanate took precedence over evidence for amoxicillin alone; consequently, fixed‐combination reports were never counted in the amoxicillin‐only group. For the strict comparative cohort, amoxicillin‐containing multicomponent products without clavulanate were excluded, whereas reports containing separately recorded eligible amoxicillin and clavulanate components were classified as amoxicillin/clavulanate.

The main analysis included reports in which the study drug was coded as primary suspect (PS) or secondary suspect (SS). Reports in which the product was recorded only as concomitant or interacting were excluded. A sensitivity analysis restricted exposure to PS reports only.

For each drug‐PT pair, a was the number of exposed reports containing the PT, b the number of exposed reports without the PT, c the number of all other deduplicated FAERS reports (excluding reports exposed to the index drug) containing the PT, and d the number of those other reports without the PT. Counts were based on unique PRIMARYID–PT pairs within a deduplicated background of 5,771,525 reports.

Adverse events were taken from the PT field of the FAERS REAC files as distributed in each quarterly public release. No external MedDRA dictionary was used to remap all quarters to a single study‐wide version; accordingly, the analysis reflects the PT coding supplied in the corresponding quarterly FAERS files. No synonym consolidation, Standardised MedDRA Query‐based recoding, or manual grouping was applied before PT‐level signal estimation. Clinical families were assigned only for post hoc narrative presentation and did not modify the underlying PT‐level calculations.

ROR was the primary signal‐detection measure and was considered positive when at least three exposed reports were available and the lower bound of the 95% confidence interval exceeded 1. PRR and IC were supportive analyses. PRR positivity required at least three exposed reports, PRR ≥ 2, and χ^2^ ≥ 4, whereas IC support required IC025 > 0. Signals were not required to satisfy all three methods simultaneously. Concordant findings were considered more robust, while method‐discordant findings were retained but interpreted cautiously.

All 3,701 distinct PTs were screened in the head‐to‐head analysis. The 19 PTs highlighted in the main manuscript were selected post hoc, after inspection of the complete PT‐level results, to provide a clinically focused summary of safety, effectiveness, and resistance‐related reporting patterns. They were not prespecified, ranked solely by effect size, or intended to represent an exhaustive set of significant findings. Of these 19 PTs, 15 met the robust comparative criteria, while four were retained for clinically relevant context and are explicitly identified as non‐robust. The selection did not affect PT‐level estimation or signal‐classification rules.

False‐positive signal proliferation was managed using established disproportionality thresholds, a minimum of three exposed reports for both ROR and PRR signal classification, triangulation across ROR, PRR, and IC, and clinical prioritization. In the head‐to‐head analysis, two‐sided Fisher exact p‐values were adjusted across all 3,701 PTs using the Benjamini–Hochberg method. A robust comparative result required a 95% confidence interval excluding 1, FDR < 0.05, and at least three reports in the group indicated by the OR direction. Complete PT‐level results are provided in the supplementary material.

For each PT, the head‐to‐head OR compared the odds that the PT was reported within the amoxicillin/clavulanate cohort versus within the amoxicillin cohort. An OR > 1 indicates a higher relative reporting proportion for amoxicillin/clavulanate, whereas an OR < 1 indicates a higher relative reporting proportion for amoxicillin. This reporting OR is not an incidence estimate and must not be interpreted as comparative clinical risk or evidence of causality.

Effectiveness‐ and resistance‐related observations were examined using five MedDRA Preferred Terms identified after the complete PT‐level screen. Drug ineffective, Treatment failure, and Condition aggravated were interpreted as nonspecific reports of reduced effectiveness or clinical worsening. Drug resistance and Pathogen resistance were considered more resistance‐specific reporting terms, although neither provides microbiological confirmation. These categories were used only for post hoc clinical interpretation and did not alter the PT‐level screening procedure.

Statistical analyses included case–non‐case disproportionality analyses based on ROR, PRR, χ^2^ statistics, and IC, together with two‐sided Fisher exact tests for the head‐to‐head comparisons. Fisher exact *p* values were adjusted for multiple testing using the Benjamini–Hochberg procedure, with FDR < 0.05 used as the predefined threshold for robust comparative findings. Analyses were performed using R version 4.5.3 (R Foundation for Statistical Computing, Vienna, Austria), with the data.table package (version 1.18.2.1) used for data management.

## 3 Results

### 3.1 Report Selection and Descriptive Characteristics

After case selection and deduplication, 5,771,525 unique FAERS reports were retained in the analytical background dataset. Among these, 10,882 reports involved amoxicillin and 10,299 involved amoxicillin/clavulanic acid as primary or secondary suspect drugs in the main analysis. The study workflow is summarized in Figure 1.

**Figure 1:**
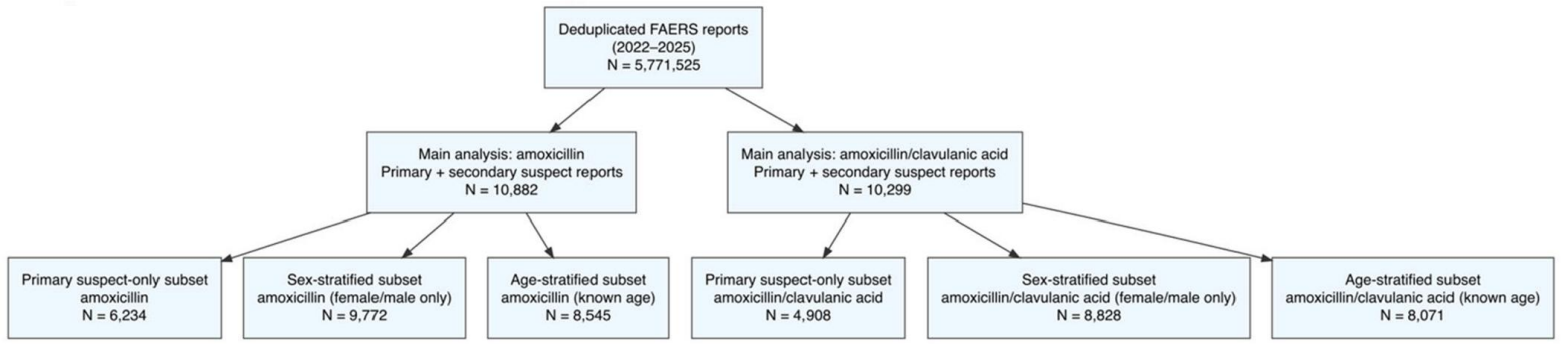
Flowchart of report selection and analytical subsets. The figure shows the deduplicated FAERS background dataset and the derivation of the main amoxicillin and amoxicillin/clavulanic acid analytical cohorts, together with the primary suspect‐only, sex‐stratified, and age‐stratified subsets.

Among amoxicillin reports, 6094 involved female patients, 3678 male patients, and 1110 reports had missing or unspecified sex. For amoxicillin/clavulanic acid, the corresponding counts were 4698, 4130, and 1471, respectively. Age distribution was mainly centered in adults aged 18–64 years, who accounted for 4499 amoxicillin reports and 4096 amoxicillin/clavulanic acid reports. Reports involving patients aged ≥ 65 years accounted for 2966 and 3243 cases, respectively, whereas pediatric reports (< 18 years) accounted for 1080 and 732 cases. Missing age information remained substantial in both groups (2337 reports for amoxicillin and 2228 for amoxicillin/clavulanic acid). Most reports were classified as serious, accounting for 9660 amoxicillin reports and 9444 amoxicillin/clavulanic acid reports. The main descriptive characteristics of the deduplicated reports are reported in Table 1.

**Table 1:** Main descriptive characteristics of deduplicated FAERS reports involving amoxicillin and amoxicillin/clavulanic acid in the primary analysis dataset.

Characteristic	Amoxicillin	Amoxicillin/clavulanic acid
Total deduplicated reports	10,882	10,299
Female	6094	4698
Male	3678	4130
Sex unknown	1110	1471
Age < 18 years	1080	732
Age 18–64 years	4499	4096
Age > = 65 years	2966	3243
Age unknown	2337	2228
Serious reports	9660	9444
Non‐serious reports	1222	855

Serious reports predominated in both exposure cohorts, accounting for 88.8% of amoxicillin reports and 91.7% of amoxicillin/clavulanic acid reports; this distribution should be interpreted as a reporting profile within FAERS rather than as an estimate of clinical incidence.

### 3.2 Main Disproportionality Findings and Clinical Signal Pattern

PT‐level disproportionality analysis identified broad but partially distinct reporting profiles for amoxicillin and amoxicillin/clavulanic acid. Overall, amoxicillin was mainly characterized by hypersensitivity‐ and cutaneous‐related signals, whereas amoxicillin/clavulanic acid showed a more pronounced hepatobiliary reporting pattern. Selected PT‐level disproportionality results, including reporting odds ratios (RORs) with 95% confidence intervals, are summarized in Table 2.

**Table 2:** Selected Preferred Term‐level disproportionality signals for amoxicillin and amoxicillin/clavulanic acid. Reporting Odds Ratios (RORs) with 95% confidence intervals are shown together with the Information Component (IC).

	Amoxicillin			Amoxicillin/clavulanic acid		
PT	*N*	ROR (95% CI)	IC	*N*	ROR (95% CI)	IC
Drug ineffective	1,151	1.778 (1.673–1.890)	0.761	836	1.327 (1.236–1.424)	0.379
Condition aggravated	260	1.220 (1.079–1.380)	0.283	552	2.831 (2.598–3.085)	1.447
Treatment failure	125	2.419 (2.027–2.887)	1.267	114	2.330 (1.936–2.803)	1.214
Pathogen resistance	15	2.406 (1.448–3.997)	1.308	54	9.298 (7.101–12.176)	3.202
Drug resistance	31	1.987 (1.396–2.828)	1.008	46	3.126 (2.338–4.179)	1.650
Diarrhea	600	1.748 (1.609–1.898)	0.770	708	2.212 (2.050–2.388)	1.088
Drug‐induced liver injury	101	4.244 (3.486–5.167)	2.073	931	48.189 (44.944–51.669)	5.351
Hepatic cytolysis	192	10.997 (9.521–12.702)	3.413	306	18.982 (16.910–21.307)	4.163
Cholestasis	94	10.158 (8.276–12.469)	3.316	277	33.467 (29.602–37.837)	4.950
Pruritus	630	2.782 (2.566–3.015)	1.418	476	2.191 (1.998–2.402)	1.093
Rash	887	3.998 (3.732–4.283)	1.902	467	2.133 (1.943–2.341)	1.056
Urticaria	536	6.587 (6.036–7.188)	2.645	312	3.952 (3.530–4.425)	1.944
Drug hypersensitivity	773	10.079 (9.363–10.851)	3.216	436	5.782 (5.251–6.367)	2.470
Drug reaction with eosinophilia and systemic symptoms	371	24.794 (22.305–27.561)	4.524	323	22.614 (20.199–25.316)	4.404
Anaphylactic reaction	371	12.380 (11.150–13.746)	3.556	246	8.519 (7.500–9.676)	3.044
Anaphylactic shock	350	27.861 (24.981–31.072)	4.687	192	15.569 (13.472–17.993)	3.902
Angioedema	423	21.958 (19.887–24.244)	4.350	160	8.360 (7.144–9.785)	3.029
Acute kidney injury	548	6.317 (5.794–6.887)	2.584	271	3.201 (2.836–3.613)	1.649
Dyspnea	636	2.447 (2.258–2.652)	1.238	630	2.569 (2.370–2.785)	1.303

*Note:* For each drug–PT pair, a (N) denotes the number of unique deduplicated reports in the exposed drug cohort containing the PT; b denotes exposed reports without the PT; c denotes all other reports in the deduplicated FAERS background containing the PT; and d denotes all other reports without the PT. The within‐drug reporting proportion was calculated as 100 × a/(a + b).

Among amoxicillin reports, the most prominent signals involved immediate hypersensitivity and cutaneous events, including anaphylactic shock (a = 350; ROR 27.861, 95% CI 24.981–31.072), anaphylactic reaction (a = 371; ROR 12.380, 95% CI 11.150–13.746), drug hypersensitivity (a = 773; ROR 10.079, 95% CI 9.363–10.851), urticaria (a = 536; ROR 6.587, 95% CI 6.036–7.188), rash (a = 887; ROR 3.998, 95% CI 3.732–4.283), and pruritus (a = 630; ROR 2.782, 95% CI 2.566–3.015). Additional relevant signals included acute kidney injury (a = 548; ROR 6.317, 95% CI 5.794–6.887), hepatic cytolysis (a = 192; ROR 10.997, 95% CI 9.521–12.702), cholestasis (a = 94; ROR 10.158, 95% CI 8.276–12.469), and dyspnea (a = 636; ROR 2.447, 95% CI 2.258–2.652).

Amoxicillin/clavulanic acid displayed a stronger hepatobiliary signature, with particularly marked disproportionality for drug‐induced liver injury (a = 931; ROR 48.189, 95% CI 44.944–51.669), cholestasis (a = 277; ROR 33.467, 95% CI 29.602–37.837), and hepatic cytolysis (a = 306; ROR 18.982, 95% CI 16.910–21.307). Hypersensitivity‐related PTs also remained prominent, including anaphylactic shock (a = 192; ROR 15.569, 95% CI 13.472–17.993), anaphylactic reaction (a = 246; ROR 8.519, 95% CI 7.500–9.676), drug hypersensitivity (a = 436; ROR 5.782, 95% CI 5.251–6.367), urticaria (a = 312; ROR 3.952, 95% CI 3.530–4.425), pruritus (a = 476; ROR 2.191, 95% CI 1.998–2.402), and rash (a = 467; ROR 2.133, 95% CI 1.943–2.341). Acute kidney injury was also detected in this group (a = 271; ROR 3.201, 95% CI 2.836–3.613), although with lower disproportionality than that observed for amoxicillin. Selected PT‐level estimates are visually summarized in Figure 2.

**Figure 2:**
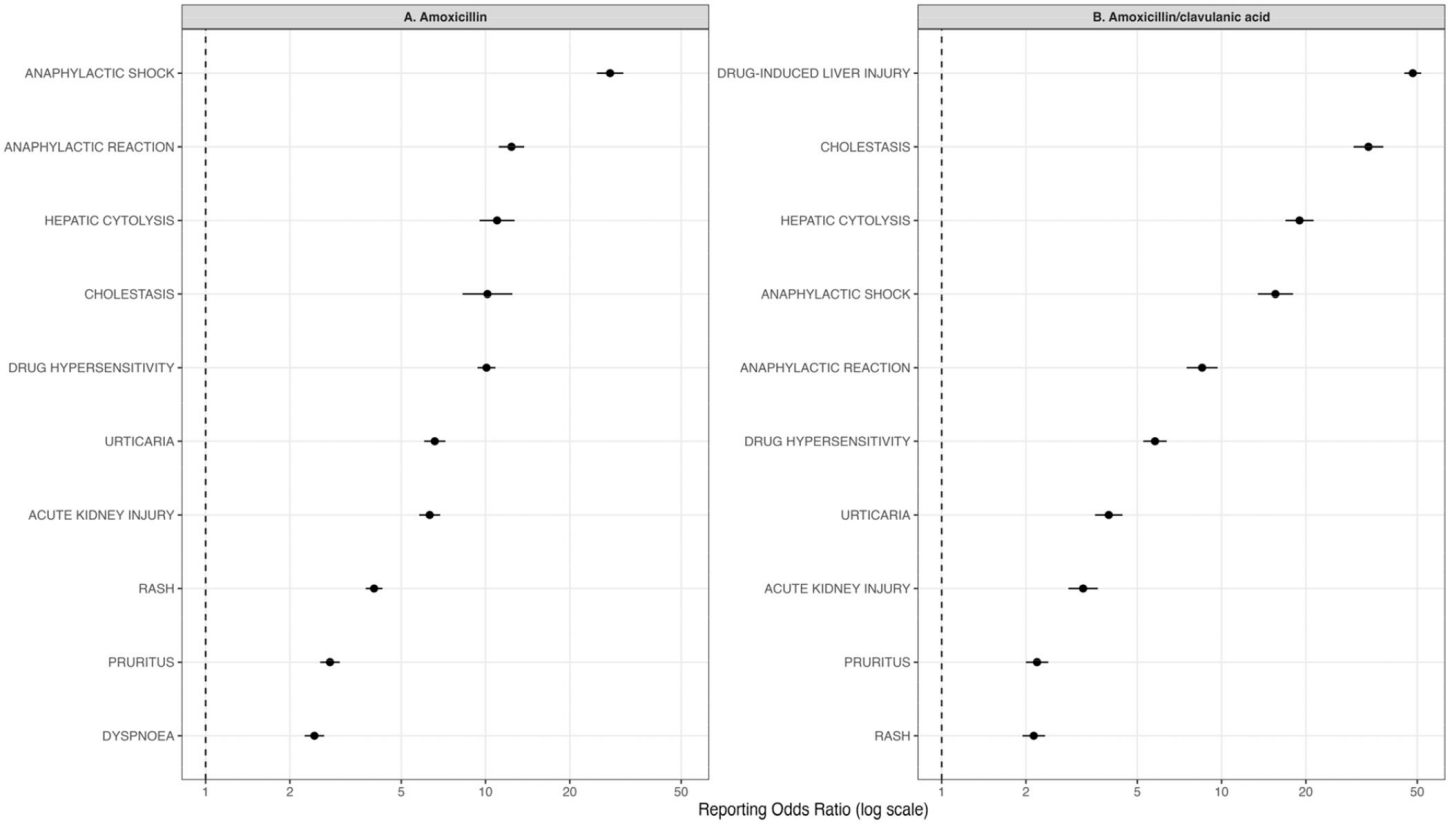
Forest plot of selected Preferred Term‐level disproportionality signals for amoxicillin and amoxicillin/clavulanic acid in the FAERS database (2022–2025). Points represent Reporting Odds Ratios (RORs), and horizontal bars indicate 95% confidence intervals. The dashed vertical line marks the null value (ROR = 1).

Comparison of the background‐based disproportionality estimates showed that the two drugs had partly divergent reporting profiles. Amoxicillin/clavulanic acid showed higher background‐based RORs for drug‐induced liver injury (48.189 vs. 4.244 for amoxicillin), cholestasis (33.467 vs. 10.158), and hepatic cytolysis (18.982 vs. 10.997). It also showed stronger disproportionality for condition aggravated (2.831 vs. 1.220), pathogen resistance (9.298 vs. 2.406), drug resistance (3.126 vs. 1.987), and diarrhea (2.212 vs. 1.748). In contrast, amoxicillin showed stronger signals for several hypersensitivity‐related PTs, including angioedema (21.958 vs. 8.360), anaphylactic shock (27.861 vs. 15.569), anaphylactic reaction (12.380 vs 8.519), drug hypersensitivity (10.079 vs. 5.782), urticaria (6.587 vs. 3.952), rash (3.998 vs. 2.133), pruritus (2.782 vs. 2.191), and acute kidney injury (6.317 vs. 3.201). Drug reaction with eosinophilia and systemic symptoms showed comparable disproportionality in both groups (24.794 vs. 22.614), while dyspnea differed only marginally (2.447 vs. 2.569).

The identical amoxicillin counts for drug reaction with eosinophilia and systemic symptoms and anaphylactic reaction were independently verified against the underlying report‐level extraction and saved analytical tables. Both PTs occurred in 371 exposed reports; however, their background counts differed, producing distinct ROR estimates of 24.794 (95% CI 22.305–27.561) and 12.380 (95% CI 11.150–13.746), respectively. These comparisons describe differences in signal magnitude relative to the FAERS background and should not be interpreted as direct comparative estimates between the two exposure cohorts; the formal head‐to‐head analysis is presented in Section 3.3.

### 3.3 Head‐to‐Head Comparison Between Amoxicillin and Amoxicillin/Clavulanic Acid

To better characterize the differences between the two exposure groups, a direct head‐to‐head comparison was performed on the shortlisted Preferred Terms shared across the main analyses. This approach was intended to complement the background‐based disproportionality analysis by identifying those PTs that were relatively more strongly represented in one exposure group than in the other. The comparative results are summarized in Table 3.

**Table 3:** Head‐to‐head comparison of selected preferred terms between amoxicillin and amoxicillin/clavulanic acid reports in FAERS.

PT	N_amox	N_amoxclav	Head‐to‐head OR (95% CI)	Comparative Interpretation
Acute kidney injury	548	271	0.510 (0.439–0.591)	Amoxicillin
Anaphylactic reaction	371	246	0.693 (0.589–0.816)	Amoxicillin
Anaphylactic shock	350	192	0.572 (0.478–0.683)	Amoxicillin
Angioedema	423	160	0.390 (0.325–0.469)	Amoxicillin
Cholestasis	94	277	3.172 (2.506–4.014)	Amoxicillin/clavulanic acid
Condition aggravated	260	552	2.314 (1.991–2.688)	Amoxicillin/clavulanic acid
Diarrhea	600	708	1.265 (1.131–1.415)	Amoxicillin/clavulanic acid
Drug hypersensitivity	773	436	0.578 (0.512–0.652)	Amoxicillin
Drug ineffective	1,151	836	0.747 (0.680–0.820)	Amoxicillin
Drug reaction with eosinophilia and systemic symptoms	371	323	0.917 (0.788–1.068)	No robust difference
Drug resistance	31	46	1.570 (0.995–2.478)	No robust difference
Drug‐induced liver injury	101	931	10.608 (8.623–13.050)	Amoxicillin/clavulanic acid
Dyspnea	636	630	1.050 (0.937–1.176)	No robust difference
Hepatic cytolysis	192	306	1.705 (1.421–2.046)	Amoxicillin/clavulanic acid
Pathogen resistance	15	54	3.819 (2.154–6.770)	Amoxicillin/clavulanic acid
Pruritus	630	476	0.789 (0.698–0.891)	Amoxicillin
Rash	887	467	0.535 (0.477–0.601)	Amoxicillin
Treatment failure	125	114	0.963 (0.746–1.243)	No robust difference
Urticaria	536	312	0.603 (0.523–0.695)	Amoxicillin

*Note:* Non‐robust comparative results are reported as “No robust difference.” A robust comparative result required a 95% CI excluding 1, FDR < 0.05, and at least three reports in the group indicated by the OR direction.

The head‐to‐head analysis confirmed that the two drugs were associated with partially divergent reporting profiles. Amoxicillin/clavulanic acid showed stronger comparative signals for hepatobiliary events, particularly drug‐induced liver injury, cholestasis, and hepatic cytolysis, supporting the more prominent hepatic reporting pattern already observed in the primary disproportionality analysis. In contrast, amoxicillin showed relatively stronger signals for several hypersensitivity‐related PTs, including angioedema, anaphylactic shock, anaphylactic reaction, drug hypersensitivity, urticaria, rash, and pruritus. Acute kidney injury was also more strongly represented in amoxicillin than in the combination group.

The direct comparison also highlighted differences in selected effectiveness‐ and resistance‐related PTs. In particular, amoxicillin/clavulanic acid showed stronger comparative signals for condition aggravated and pathogen resistance, whereas drug resistance did not meet the predefined criteria for a robust head‐to‐head difference. Overall, the head‐to‐head findings were consistent with the broader signal architecture observed in the background‐based analyses and provided a more focused comparative view of the distinctive reporting patterns associated with the two treatments.

### 3.4 Secondary and Exploratory Analyses

Several secondary and exploratory analyses were performed to assess the robustness and clinical consistency of the main findings. Overall, these analyses did not materially modify the primary signal architecture.

Restriction to primary suspect reports reduced the total number of detectable signals while preserving the core pattern. In the PS‐only analysis, 460 ROR signals and 422 PRR signals were retained for amoxicillin, compared with 401 ROR and 384 PRR signals for amoxicillin/clavulanic acid; 426 and 375 main ROR signals, respectively, remained stable under this restriction.

Sex‐stratified analyses showed that the main shortlisted PTs were generally preserved in both females and males. In the amoxicillin group, 7 of 12 shortlisted PTs showed higher ROR values in females and 5 in males, with 6 PTs showing non‐overlapping confidence intervals; in the amoxicillin/clavulanic acid group, the corresponding distribution was 5 and 7 PTs, with only 3 showing non‐overlapping confidence intervals.

Age‐stratified analyses revealed clinically coherent heterogeneity. For amoxicillin, urticaria was most prominent in patients aged < 18 years (ROR = 10.818), whereas acute kidney injury was strongest in older patients (ROR = 9.155). For amoxicillin/clavulanic acid, hepatobiliary PTs increased markedly with age, with drug‐induced liver injury rising from ROR = 10.500 in pediatric reports to 23.109 in older patients, and cholestasis from 1.891 to 43.989.

Exploratory burden analyses suggested that the main PT signals were not confined to highly complex reports, although signal strength tended to attenuate with increasing therapeutic complexity. A curated analysis of effectiveness‐ and resistance‐related PTs identified additional patterns, including drug ineffective (a = 1,151; ROR = 1.778) and treatment failure (a = 125; ROR = 2.419) for amoxicillin, and condition aggravated (a = 552; ROR = 2.831), drug resistance (a = 46; ROR = 3.126), and pathogen resistance (a = 54; ROR = 9.298) for amoxicillin/clavulanic acid. These findings were interpreted as exploratory pharmacovigilance signals rather than as direct microbiological evidence of antimicrobial resistance.

## 4 Discussion

This pharmacovigilance analysis provides a comprehensive real‐world assessment of the safety profile and potential signals of reduced therapeutic effectiveness associated with amoxicillin and amoxicillin/clavulanic acid, based on FAERS data collected between 2022 and 2025. The findings delineate a coherent pattern in which safety outcomes align with established pharmacological knowledge, while also identifying a set of effectiveness‐ and resistance‐related PTs that may reflect emerging challenges in antimicrobial performance.

A prominent feature of the present analysis is the overwhelming predominance of serious reports for both treatments, with a markedly higher serious‐to‐non‐serious ratio observed for the combination of amoxicillin and clavulanic acid. At first glance, this might suggest a less favorable safety profile for the combination therapy; however, such an interpretation would be overly simplistic.

A possible explanation is that, in routine clinical practice, amoxicillin/clavulanic acid is frequently prescribed for patients with more complex infections or in situations where broader antimicrobial coverage is considered appropriate, including cases in which antimicrobial resistance is suspected or previous antibiotic therapy has been unsuccessful. This prescribing pattern has been described in previous studies and may contribute to confounding by indication. Accordingly, the observed imbalance is more likely to reflect differences in clinical prescribing context and reporting behavior within FAERS than intrinsic differences in drug safety [4].

The qualitative distribution of adverse events further supports the internal consistency of the dataset. Reports associated with amoxicillin were predominantly characterized by hypersensitivity and cutaneous manifestations, including rash, pruritus, urticaria, and related allergic PTs, which are well‐recognized features of β‐lactam antibiotics. These findings are complemented by the presence of systemic symptoms and by less frequent but clinically relevant events such as acute kidney injury, in line with recent literature drawing renewed attention to amoxicillin‐associated nephrotoxicity [14, 27]. In contrast, the safety profile of amoxicillin/clavulanic acid was distinguished by a stronger hepatobiliary signature, particularly for drug‐induced liver injury, cholestasis, and hepatic cytolysis. This pattern is coherent with the known hepatic liability of the combination and is consistent with previous literature identifying clavulanic acid as a major contributor to this aspect of its safety profile [15, 28, 29].

The direct head‐to‐head comparison further strengthened this interpretation. While both treatments shared part of their safety spectrum, the comparative analysis confirmed that amoxicillin/clavulanic acid was more strongly characterized by hepatobiliary PTs, whereas amoxicillin showed relatively stronger signals for several hypersensitivity‐related PTs and for acute kidney injury. This finding is important because it refines the background‐based disproportionality analysis and helps distinguish PTs that are common to both drugs from those that more clearly define one exposure profile. Overall, the head‐to‐head results support the view that the two treatments are associated with partially divergent reporting patterns rather than a simple difference in signal magnitude.

The secondary analyses added further consistency to the main findings. Restriction to primary suspect reports reduced the overall number of detectable signals, as expected, but preserved the core signal architecture in both exposure groups. Similarly, the sex‐ and age‐stratified analyses showed heterogeneity that was clinically plausible rather than contradictory. In particular, the age‐related increase of hepatobiliary PTs in the amoxicillin/clavulanic acid group is in line with previous clinical observations suggesting that this type of liver injury is more often recognized in older patients [15, 28]. Exploratory analyses based on concomitant‐drug burden pointed in the same direction, suggesting that the principal PT signals were not confined to the most complex reports, although signal strength tended to attenuate as therapeutic complexity increased.

Beyond the characterization of safety outcomes, the present study also identified a set of Preferred Terms related to reduced therapeutic effectiveness, including drug ineffective, treatment failure, and condition aggravated, together with more specific resistance‐related Preferred Terms such as pathogen resistance and drug resistance. These categories should not be considered equivalent. The former represent broad clinical outcomes that may arise from multiple factors, including disease severity, inappropriate antibiotic selection, suboptimal dosing, poor adherence, or diagnostic uncertainty, whereas the latter may more specifically suggest concerns regarding antimicrobial resistance but still lack microbiological confirmation within the FAERS database. Consequently, none of these Preferred Terms should be interpreted as direct evidence of antimicrobial resistance or reduced antimicrobial effectiveness. Rather, their recurrent identification represents exploratory pharmacovigilance signals that may warrant further clinical and microbiological investigation.

This interpretation is consistent with the growing view that pharmacovigilance systems may complement conventional antimicrobial surveillance by generating hypotheses based on reporting patterns that deserve further clinical and microbiological evaluation [21]. Comparative effectiveness data from clinical settings should therefore be considered complementary rather than directly interchangeable with spontaneous reporting patterns [30].

The demographic characteristics observed in this study further contextualize the findings. The predominance of adult and elderly patients is consistent with the epidemiology of infections commonly treated with amoxicillin‐based therapies, while the higher representation of females in amoxicillin‐related reports aligns with previously described differences in healthcare utilization and reporting behavior. At the same time, the substantial proportion of cases with missing demographic information highlights a persistent limitation of spontaneous reporting systems and introduces an additional layer of uncertainty in subgroup analyses.

Several limitations inherent to the FAERS database must be considered when interpreting these findings. The voluntary nature of reporting introduces the possibility of underreporting and reporting bias, with a tendency toward the submission of more severe or unusual cases. The absence of denominator data precludes the estimation of incidence rates and limits quantitative risk assessment.

Moreover, key clinical variables such as antibiotic dose, route of administration, treatment duration, and microbiological susceptibility testing are not systematically available in FAERS, further limiting the interpretation of reports suggestive of reduced therapeutic effectiveness or antimicrobial resistance.

Furthermore, the lack of detailed clinical and microbiological information, including pathogen identification and susceptibility profiles, prevents direct attribution of therapeutic failure to antimicrobial resistance. Additional factors such as inappropriate prescribing, suboptimal dosing, poor adherence, or diagnostic uncertainty may also contribute to reports of reduced effectiveness and cannot be disentangled within the constraints of the available data. Confounding by indication is also particularly relevant in this setting, especially for amoxicillin/clavulanic acid, which may be preferentially used in more complex infections or in cases where resistance is already suspected.

Despite these limitations, the present study provides valuable real‐world insights into the reporting profiles of two widely used antibiotics. The convergence of safety signals consistent with established pharmacology, the clearer distinction between the two drugs provided by the head‐to‐head analysis, and the recurrent identification of Preferred Terms related to reduced therapeutic effectiveness or resistance‐related reporting contexts support the value of pharmacovigilance databases as complementary tools for safety characterization and hypothesis generation. However, these reporting signals require independent clinical and microbiological validation before any conclusions regarding antimicrobial effectiveness or resistance can be drawn.

## 5 Conclusion

Overall, this study highlights the potential value of pharmacovigilance data as an underused resource for identifying hypothesis‐generating reporting signals related not only to drug safety but also to patterns that may warrant further investigation regarding therapeutic effectiveness. In this FAERS‐based analysis, amoxicillin and amoxicillin/clavulanic acid showed partially overlapping but distinct reporting profiles, with hypersensitivity‐related signals more prominent for amoxicillin and hepatobiliary signals more evident for the combination. The recurrent identification of effectiveness‐ and resistance‐related Preferred Terms should not be interpreted as evidence of reduced antimicrobial effectiveness or microbiologically confirmed antimicrobial resistance, as these reports may reflect a variety of clinical and therapeutic factors, including nonadherence, inappropriate antibiotic selection, inadequate dosing, disease severity, or diagnostic uncertainty. Rather, these findings represent exploratory pharmacovigilance signals that may complement conventional surveillance systems and generate hypotheses for further investigation. Independent clinical, microbiological, and pharmacoepidemiological studies are essential to validate these observations and to better define the role of spontaneous reporting systems in monitoring antibiotic use and performance.

## Author Contributions

**Pierangela Irrera:** methodology, resources. **Ilaria Ammendolia:** methodology, formal analysis. **Gabriella Frattima:** methodology. **Fabiola De Luca:** methodology. **Michela Campolo:** supervision. **Emanuela Esposito:** conceptualization, supervision. **Alessio Ardizzone:** conceptualization, writing – original draft. **Fabrizio Calapai:** writing – review and editing, software.

## Funding

The authors have nothing to report.

## Conflicts of Interest

The authors declare no conflicts of interest.

## Declaration of Generative AI and AI‐Assisted Technologies

Generative AI was not used in this manuscript; all scientific content was independently reviewed and verified by the authors, who take full responsibility for the final manuscript.

## Transparency Statement

The corresponding author (Emanuela Esposito) affirms that this manuscript is an honest, accurate, and transparent account of the study being reported; that no important aspects of the study have been omitted; and that any discrepancies from the study as planned (and, if relevant, registered) have been explained.

## Supporting information


Supporting File

## Data Availability

The authors confirm that the data supporting the findings of this study are available within the article and its supplementary materials.
