# Supplementary material for: Comparative Adverse Event Reporting Patterns of Amoxicillin and Amoxicillin/Clavulanic Acid in FAERS: A 2022–2025 Disproportionality Analysis
Source: Health Sci Rep. 2026 Oct 5;9(10):e73328. doi: 10.1002/hsr2.73328 (PMC13635800; doi:10.1002/hsr2.73328)
Supplement: Supplementary file 1 [file HSR2-9-e73328-s001.docx]

# Supplementary Table 1

Audit list of the 42 normalized source expressions recovered from DRUGNAME and PROD_AI. Frequency indicates the number of source rows carrying the corresponding combined expression in the drug-capture audit. Rule codes: A = eligible amoxicillin-only evidence; C = amoxicillin/clavulanate combination evidence; X = amoxicillin plus a non-clavulanate active ingredient, excluded from the strict amoxicillin-only head-to-head cohort; S = clavulanate-containing expression without amoxicillin, not sufficient alone and evaluated only in report-level component pairing.

| **No.** | **Normalized captured expression** | **Rule** | **Frequency** |
| --- | --- | --- | --- |
| 1 | AMOXICILLIN AMOXICILLIN | A | 12,563 |
| 2 | AMOXICILLIN + CLAVULANIC ACID AMOXICILLIN + CLAVULANIC ACID | C | 5,173 |
| 3 | AMOXICILLIN + CLAVULANATE POTASSIUM AMOXICILLIN + CLAVULANATE POTASSIUM | C | 3,654 |
| 4 | AMOXICILLIN + CLAVULANATE POTASSIUM AUGMENTIN | C | 2,359 |
| 5 | AMOXICILLIN AMOXICILLIN TRIHYDRATE | A | 1,511 |
| 6 | AMOXICILLIN + CLAVULANATE POTASSIUM AMOXICILLIN AND CLAVULANATE POTASSIUM | C | 446 |
| 7 | CLAVULANIC ACID CLAVULANIC ACID | S | 360 |
| 8 | AMOXICILLIN SODIUM AMOXICILLIN SODIUM | A | 208 |
| 9 | AMOXICILLIN + OMEPRAZOLE MAGNESIUM + RIFABUTIN TALICIA | X | 153 |
| 10 | CLAVULANATE POTASSIUM CLAVULANATE POTASSIUM | S | 121 |
| 11 | AMOXICILLIN + CLARITHROMYCIN + ESOMEPRAZOLE AMOXICILLIN + CLARITHROMYCIN + ESOMEPRAZOLE | X | 87 |
| 12 | AMOXICILLIN POLYMOX | A | 42 |
| 13 | AMOXICILLIN AMOXIL | A | 35 |
| 14 | AMOXICILLIN + CLARITHROMYCIN + VONOPRAZAN FUMURATE AMOXICILLIN + CLARITHROMYCIN + VONOPRAZAN FUMURATE | X | 28 |
| 15 | AMOXICILLIN + CLARITHROMYCIN + VONOPRAZAN FUMARATE VOQUEZNA TRIPLE PAK | X | 21 |
| 16 | AMOXICILLIN + CLARITHROMYCIN + OMEPRAZOLE AMOXICILLIN + CLARITHROMYCIN + OMEPRAZOLE | X | 16 |
| 17 | AMOXICILLIN + CLARITHROMYCIN + LANSOPRAZOLE PREVPAC | X | 15 |
| 18 | AMOXICILLIN + CLAVULANATE POTASSIUM AUGMENTIN XR | C | 14 |
| 19 | AMOXICILLIN + VONOPRAZAN FUMARATE VOQUEZNA DUAL PAK | X | 11 |
| 20 | AMOXICILLIN + CLAVULANIC ACID BIOCLAVID | C | 9 |
| 21 | AMOXICILLIN + CLARITHROMYCIN + LANSOPRAZOLE AMOXICILLIN + CLARITHROMYCIN + LANSOPRAZOLE | X | 7 |
| 22 | AMOXICILLIN + CLARITHROMYCIN + PANTOPRAZOLE AMOXICILLIN + CLARITHROMYCIN + PANTOPRAZOLE | X | 6 |
| 23 | AMOXICILLIN + SULBACTAM AMOXICILLIN + SULBACTAM | X | 6 |
| 24 | AMOXICILLIN + CLOXACILLIN AMOXICILLIN + CLOXACILLIN | X | 5 |
| 25 | AMOXICILLIN AMOXICILLIN 500 MG | A | 5 |
| 26 | AMOXICILLIN + BROMHEXINE AMOXICILLIN + BROMHEXINE | X | 5 |
| 27 | AMOXICILLIN + FLUCLOXACILLIN AMOXICILLIN + FLUCLOXACILLIN | X | 4 |
| 28 | CEFPODOXIME + CLAVULANIC ACID CEFPODOXIME + CLAVULANIC ACID | S | 4 |
| 29 | AMOXICILLIN + CLARITHROMYCIN + RABEPRAZOLE AMOXICILLIN + CLARITHROMYCIN + RABEPRAZOLE | X | 2 |
| 30 | AMOXICILLIN + SULBACTAM PIVOXIL AMOXICILLIN + SULBACTAM PIVOXIL | X | 2 |
| 31 | AMOXICILLIN + CLARITHROMYCIN + OMEPRAZOLE OMECLAMOX-PAK | X | 2 |
| 32 | AMOXICILLIN AMOXICILLAN | A | 2 |
| 33 | CLAVULANIC ACID + TICARCILLIN CLAVULANIC ACID + TICARCILLIN | S | 2 |
| 34 | CLAVULANATE POTASSIUM + TICARCILLIN DISODIUM CLAVULANATE POTASSIUM + TICARCILLIN DISODIUM | S | 2 |
| 35 | CEFPODOXIME PROXETIL + CLAVULANATE POTASSIUM CEFPODOXIME PROXETIL + CLAVULANATE POTASSIUM | S | 2 |
| 36 | AMPICILLIN TRIHYDRATE + CLAVULANATE POTASSIUM AMPICILLIN TRIHYDRATE + CLAVULANATE POTASSIUM | S | 2 |
| 37 | AMOXICILLIN + METRONIDAZOLE + RABEPRAZOLE AMOXICILLIN + METRONIDAZOLE + RABEPRAZOLE | X | 1 |
| 38 | AMOXICILLIN + DICLOXACILLIN SODIUM AMOXICILLIN + DICLOXACILLIN SODIUM | X | 1 |
| 39 | AMOXICILLIN + CLAVULANATE POTASSIUM AUGMENTIN ES-600 | C | 1 |
| 40 | CLAVULANIC ACID + TICARCILLIN DISODIUM CLAVULANIC ACID + TICARCILLIN DISODIUM | S | 1 |
| 41 | CEFIXIME + CLAVULANATE POTASSIUM CEFIXIME + CLAVULANATE POTASSIUM | S | 1 |
| 42 | AMOXICILLIN + SERRAPEPTASE AMOXICILLIN + SERRAPEPTASE | X | 1 |
